# Multifunctional Applications of Lactic Acid Bacteria: Enhancing Safety, Quality, and Nutritional Value in Foods and Fermented Beverages

**DOI:** 10.3390/foods13233714

**Published:** 2024-11-21

**Authors:** Christian Kosisochukwu Anumudu, Taghi Miri, Helen Onyeaka

**Affiliations:** School of Chemical Engineering, University of Birmingham, Birmingham B15 2TT, UK; cka329@student.bham.ac.uk (C.K.A.); t.miri@bham.ac.uk (T.M.)

**Keywords:** lactic acid bacteria, starter cultures, food safety, probiotics, bacteriocins, food quality

## Abstract

Lactic Acid Bacteria (LAB) have garnered significant attention in the food and beverage industry for their significant roles in enhancing safety, quality, and nutritional value. As starter cultures, probiotics, and bacteriocin producers, LAB contributes to the production of high-quality foods and beverages that meet the growing consumer demand for minimally processed functional and health-promoting food products. Industrial food processing, especially in the fresh produce and beverage sector, is shifting to the use of more natural bioproducts in food production, prioritizing not only preservation but also the enhancement of functional characteristics in the final product. Starter cultures, essential to this approach, are carefully selected for their robust adaptation to the food environment. These cultures, often combined with probiotics, contribute beyond their basic fermentation roles by improving the safety, nutritional value, and health-promoting properties of foods. Thus, their selection is critical in preserving the integrity, quality, and nutrition of foods, especially in fresh produce and fruits and vegetable beverages, which have a dynamic microbiome. In addition to reducing the risk of foodborne illnesses and spoilage through the metabolites, including bacteriocins they produce, the use of LAB in these products can contribute essential amino acids, lactic acids, and other bioproducts that directly impact food quality. As a result, LAB can significantly alter the organoleptic and nutritional quality of foods while extending their shelf life. This review is aimed at highlighting the diverse applications of LAB in enhancing safety, quality, and nutritional value across a range of food products and fermented beverages, with a specific focus on essential metabolites in fruit and vegetable beverages and their critical contributions as starter cultures, probiotics, and bacteriocin producers.

## 1. Introduction

Lactic acid bacteria (LAB) have long been crucial in the production of fermented foods and beverages, serving as natural agents for preservation, flavor enhancement, and nutritional improvement. They are Gram-positive, non-spore-forming, non-pathogenic bacteria, typically anaerobic or facultative aerobic, that metabolize carbohydrates to produce lactic acid during fermentation [1]. With a rich history rooted in ancient food processing techniques, LAB’s relevance in modern food science has only grown, reflecting its multifaceted role in addressing contemporary challenges in food safety, quality, and sustainability [2]. These microorganisms, often part of the natural microbiota or intentionally added as starter cultures, initiate fermentation processes that not only extend the shelf life of perishable foods but also introduce desirable sensory characteristics and enhanced nutritional profiles [3]. Their ability to utilize various substrates and produce a wide range of metabolites, such as organic acids, bacteriocins, and other antimicrobial compounds, makes them essential for enhancing food safety, flavor, and preservation [4]. Foodborne illnesses have become a significant global health threat, affecting over 600 million people annually [5]. These diseases are caused by a wide range of pathogenic microorganisms such as *Salmonella*, *Campylobacter*, and *E. coli* that, at one stage or another, contaminate food during production, processing, storage, and distribution, threatening not only public health but also the global economy [6,7]. LAB play a crucial role in mitigating these risks in fermented foods and beverages by acting as natural preservatives and inhibiting the growth of harmful microorganisms [3]. Like other food products, fermented foods and beverages have safety concerns that require a proper understanding of the process of fermentation involved and the microbial activity occurring to avoid health risks. Safety in fermented foods depends largely on maintaining the right balance of beneficial microorganisms during the fermentation process [8]. If the environment for fermentation is not optimal, the beneficial microbes may not be dominant, and contamination with harmful bacteria, molds, or toxins can occur [9].

LAB are of great importance to the fermentation process due to their contribution through a unique enzymatic system that produces extra-cellular protease and extra-cellular lipase enzymes that hydrolyze proteins, fats, and carbohydrates into smaller molecules [10]. Species of *Lactiplantibacillus*, *Lactobacillus*, *Leuconostoc*, and *Pediococcus*, produce enzymes during fermentation that can hydrolyze numerous food components like carbohydrates and lipids into flavor precursors that can be converted into aroma compounds with complex sensory profiles [11,12]. They also contribute to the textural development of many fermented products, such as yogurt, cheese, sourdough bread, and pickled vegetables, through their metabolic activities such as acidification, exopolysaccharide production, and enzymatic reactions [11,13]. With the advent of traditional food preservation techniques, such as the addition of chemical preservatives posing health risks, biopreservation techniques, particularly those involving LAB and their metabolites, are becoming more favorable for improving food quality, preservation of color, and freshness [14]. During fermentation, LAB release a variety of bioactive compounds and signal molecules that can positively influence human health. These bacteria and their metabolites interact with the gut microbiome to maintain intestinal and overall body health [15]. Although many essential nutrients, such as vitamins and minerals, are present in a wide variety of foods, their bioavailability, or the extent to which the body can absorb and utilize them, is often limited. Factors like the presence of antinutritional compounds (e.g., phytates and oxalates) in plant-based foods can bind to these nutrients, reducing their absorption in the body [16]. LAB enhance the bioavailability of these essential nutrients by breaking down complex compounds, making them easier for the body to absorb [17]. LAB, particularly strains from genera like *Lactobacillus* and *Lactiplantibacillus*, are widely recognized for their probiotic properties, offering a multitude of health benefits when consumed in fermented foods and beverages [18]. The functional compounds synthesized by probiotic LAB strains include some essential vitamins, such as folate, riboflavin (B2), and B12, and short-chain fatty acids (SCFAs), which play important functions like promoting gut health and improving the general nutritional profile of fermented products [19].

LAB are an essential component of the traditional food fermentation process, which has been utilized for generations as a natural means of preserving and enhancing food [2]. In fermented foods with dairy ingredients, like yogurts, kefirs, and cheeses, LAB are an essential factor not only in fermentation but also in the improvement of safety and taste. They secrete organic acids that impede the growth of pathogenic microorganisms while giving a normal sour taste to the product [3]. With the rapid growth in the consumption of fermented beverages, especially as health-conscious consumers increasingly view them as refreshing, convenient, and nutritious, mostly due to their bioactive compound content and purported probiotic effect, LAB play a crucial role in improving their quality [20]. However, the selection and stability of strains are two important success factors to consider when applying LAB in food and beverages. The immense variability in the various products and environments in which they are expected to function poses a challenge when selecting LAB strains for use [21]. The composition, pH, water activity, and conditions of processing for fermented foods and beverages vary greatly and can affect the growth, metabolic activity, and viability of the strains of LAB [22]. It is, therefore, important to select strains that will perform well under product conditions. Regulatory challenges and consumer perceptions continue to influence the integration of LAB into the food market, especially when it comes to labeling them as probiotics or natural preservatives [23]. While bacteriocins produced by LAB, such as nisin from *L. lactis*, have gained acceptance and regulatory approvals for use as preservatives in certain regions of the world, approval for the use of many other bacteriocins has not been granted as approvals for new compounds derived from LAB is an elaborate process [24]. Growing consumer awareness and demand for clean-label ingredients have led to increasing skepticism toward artificial additives and preservatives, driving interest in natural alternatives like LAB [23].

LAB have also become a promising route to address the challenges of food waste by providing new methods of reducing food loss as well as promoting sustainable food production [25]. Since sustainability in food production also involves the reduction in synthetic chemicals, which can have severe consequences in terms of health and environmental impact, LAB fermentation represents a natural alternative to chemical preservatives and additives that are used so often in a wide variety of food products [23]. This review explores the multifunctional applications of LAB, focusing on their contributions to food safety, quality enhancement, and nutritional improvements.

### 1.1. Overview of Lactic Acid Bacteria (LAB) and Their Characteristics

**Figure 1:**
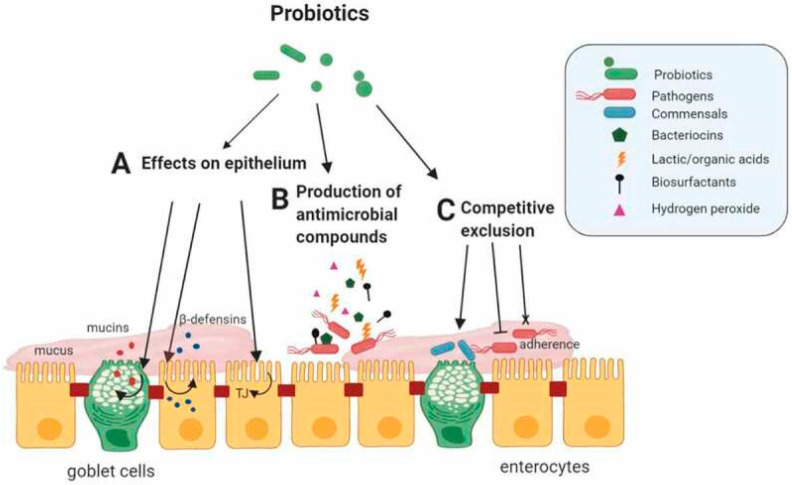
Mechanism of probiotic LAB strains against enteric pathogens in the gut [32].

LAB are Gram-positive, non-spore-forming, non-pathogenic bacteria, typically anaerobic or facultative aerobic, that metabolize carbohydrates to produce lactic acid during fermentation [1]. They are ubiquitous in nature and can be found in a wide range of environments, commonly in decaying plants, animal matter, and various raw materials used in fermented foods, as well as in the gut of herbivores and humans [26]. In the gut, LAB form a symbiotic relationship with their hosts, where they help maintain a balanced microbiome by inhibiting the growth of harmful pathogens through competitive exclusion and the production of antimicrobial substances, including bacteriocins [27]. Figure 1 presents the mechanism used by probiotic LAB strains to protect the gut against enteric infections.

LAB have been extensively used in the food industry for over 6000 years, where they have been employed in the fermentation of dairy products such as yogurt and cheese; meat products like sausages; and plant-based foods such as sauerkraut, kimchi, and sourdough bread [2]. Their ability to preserve food through lactic acid production has been essential in extending the shelf life of perishable products in the absence of refrigeration. Their ability to adapt to various environments, from food products like dairy and meats to human mucosal surfaces, is due to their unique nutritional, environmental, and adhesional properties, which allows them to play a crucial role in various ecological niches, particularly in the production of fermented foods and beverages [28].

With the ability of LAB to convert carbohydrates into lactic acid during fermentation, they aid in the preservation of food and the enhancement of its flavor and nutritional value [29]. Based on their fermentation pathways, LAB are classified into two main groups: homofermentative and heterofermentative. Homofermentative LAB, such as *Lactococcus* and *Streptococcus*, primarily produce lactic acid as the sole end product of fermentation, converting one molecule of glucose into two molecules of lactate [1,26]. In contrast, heterofermentative LAB, such as *Leuconostoc* and *Weissella*, follow a more complex metabolic pathway, producing a mixture of by-products, including lactate, ethanol, and carbon dioxide, from the fermentation of a single glucose molecule [1,30]. This distinction between the two groups is significant, as it influences the characteristics of the fermentation process, including the flavor profile, texture, and gas production in food products [29]. Homofermentative LAB are often favored in processes where high lactic acid yield is desired, while heterofermentative LAB contribute to more diverse fermentation outcomes that are useful in the production of foods like sourdough bread and certain cheeses [30,31].

### 1.2. Importance of LAB in Food Processing (Production, Fermentation, Quality Improvement and Safety)

LAB play a critical role in food processing, especially in the production and fermentation of various foods, ensuring that they are safe and of high quality. In food production, LAB are indispensable as they are responsible for the fermentation of dairy products such as yogurt, cheese, and kefir, as well as fermented vegetables like sauerkraut, kimchi, and pickles [33]. LAB are also central to the production of fermented meat products, such as salami, and plant-based products like sourdough bread [34]. During the fermentation process, LAB convert sugars (mainly glucose) present in raw materials into lactic acid. This lactic acid production not only preserves the food by creating an acidic environment that inhibits spoilage organisms but also transforms the raw ingredients into products with enhanced flavor and texture [3,34]. LAB play a key role in preventing staling in bakery products by producing lactic acid, which helps retain moisture and improve the texture of bread. During the staling process, amylose and amylopectin in starch undergo recrystallization, causing the bread to harden and lose freshness [35]. LAB fermentation delays this process by modifying the starch structure and enhancing the crumb softness, thereby extending the shelf life of the bread and maintaining its palatability over time [36].

LAB play a crucial role in improving food safety by producing antimicrobial compounds such as lactic acid, acetic acid, and antifungal substances during fermentation. These compounds inhibit the growth of harmful microorganisms, thereby preserving the freshness and extending the shelf life of baked goods [37]. LAB fermentation also helps reduce the formation of acrylamide, a toxic byproduct found in carbohydrate-rich foods like baked bread. Studies like that of Nachi et al. [38] have shown that certain LAB strains of *L. brevis*, *L. plantarum*, and *Pediococcus pentosaceus* can significantly lower acrylamide levels in bread. Additionally, LAB strains of *L. plantarum* that produce anti-mold compounds are increasingly sought after to further extend the shelf life of bakery products, considering the role they play in detoxifying harmful mycotoxins and preventing fungal growth through enzymatic activity, adsorption, and competition with fungi [39]. By producing proteolytic enzymes, such as cell-wall proteinases and peptide transporters, LAB can degrade mycotoxins into less harmful substances, while citric acid and other organic acids produced during fermentation help break down aflatoxins [36].

LAB have been identified as the dominant microorganisms in many fermented foods, playing a critical role in their production due to their unique physiological and metabolic traits [33]. Their ability to utilize various substrates and produce a wide range of metabolites, such as organic acids, bacteriocins, and other antimicrobial compounds, makes them essential for enhancing food safety, flavor, and preservation [4]. Their metabolic processes, particularly lactic acid fermentation, not only inhibit spoilage and pathogenic microorganisms but also improve the sensory qualities of fermented foods, such as texture, taste, and aroma [3]. LAB also plays a significant role in the fermentation of traditional foods across various cultures, contributing to their unique flavors, textures, and preservation. In Turkish cuisine, tarhana, a fermented cereal-based soup mix made from grains, vegetables, and yogurt, undergoes lactic acid fermentation, giving it its characteristic sour taste, with LAB species such as *L. plantarum*, *S. thermophilus*, and *Weissella cibaria* being instrumental in determining its sensory properties [40]. Similarly, in the Middle Eastern drink boza, made from grains like millet or wheat, LAB fermentation converts starches into sugars and lactic acid, resulting in a sweet and sour flavor [36]. *L. paracasei*, *L. plantarum*, and *L. lactis* are commonly identified in boza, contributing to its distinct taste and texture [41]. In Asian cuisine, stinky tofu is fermented in a brine solution, where LAB, along with species from the *Bacillus* genus, dominate the microbial community, giving the tofu its signature odor and flavor [4]. Common types of LAB and their scope of application in food processing are presented in Table 1.

### 1.3. Metabolites of LAB

LAB are well-known for their ability to ferment carbohydrates into lactic acid, a process central to the preservation, safety, and sensory quality of fermented foods [29]. However, the metabolic capabilities of LAB extend far beyond lactic acid production. LAB synthesize a wide array of metabolites, including organic acids, antimicrobial peptides, exopolysaccharides, vitamins, enzymes, and bioactive compounds [42,43]. These metabolites not only contribute to food preservation and flavor enhancement but also offer significant health benefits, making LAB highly significant in both the food industry and probiotic development. Table 2 presents some of the metabolites synthesized by LAB and their primary functions.

#### 1.3.1. Bacteriocins

Bacteriocins are proteinaceous compounds produced by many LAB species, which exhibit antimicrobial activity, either targeting closely related species (narrow spectrum) or a broad range of microorganisms (broad spectrum) [47]. Bacteriocins were previously categorized into four main classes based on their structural characteristics and properties: class I, which includes modified peptides, such as lantibiotics like nisin, which contain unusual amino acids such as lanthionine and are often antibiotic-like; class II, which consists of unmodified, small, heat-stable peptides (less than 10 kDa) that are linear and do not contain lanthionine; class III, which encompasses larger, heat-sensitive peptides (over 30 kDa) that are less stable under high temperatures; and class IV, which includes small, circular peptides (less than 10 kDa) with unique structural features [42,48]. However, this classification scheme has evolved into broadly three classes, with the large complexes with carbohydrate or lipid moieties being categorized as bacteriolysins and comprised of leuconosin S and lactocin 27 [49]. In this new scheme, Class I bacteriocins are further divided into the lantibiotics (Class 1a), the labyrinthopeptins (Class 1b), and the sanctibiotics (Class 1c) [50]. Similarly, Class II bacteriocins are subdivided into pediocin-like bacteriocins (Class IIa) and unmodified bacteriocins (Class IIb), while larger circular peptides are now classified as Class IIb bacteriocins and the last sub-group (Class IId) comprising of unmodified, linear, and non-pediocin-like bacteriocins [51,52,53]. Each strain of LAB produces its own specific bacteriocin, differing in their mode of action, molecular size, genetic origin, and biochemical characteristics, enabling a diverse range of antimicrobial activities across different microbial targets [54].

The most well-known bacteriocin, nisin, produced by *L. lactis*, is commercially approved as a food preservative by regulatory bodies like the FDA and EFSA in dairy products, canned foods, and beverages due to its effectiveness in extending shelf life and enhancing food safety [24]. The antimicrobial activity of nisin is primarily due to its ability to disrupt the integrity of bacterial cell membranes by forming pores, leading to leakage of cellular contents and cell death [55]. In addition, nisin inhibits cell wall biosynthesis by binding to lipid II, a key molecule in bacterial cell wall construction, preventing the synthesis of peptidoglycan, which is essential for cell wall integrity [56]. This dual action makes nisin highly effective against a range of Gram-positive bacteria, including foodborne pathogens such as *Listeria monocytogenes*, making it an ideal preservative in fermented foods [55]. Other bacteriocins that have been discovered and used in the food industry in recent decades include *Leuconostoc lactis* SM2, which produces Leucocin that exhibits strong antimicrobial activity, particularly against bacteria such as *L. monocytogenes* [42], and *Lactiplantibacillus plantarum* NWAFU-BIO-BS29, which produces Plantaricin Bio-LP1, a potent bio-preservative that effectively inhibits a wide range of harmful pathogens, including *Escherichia coli*, *Staphylococcus aureus*, and *Salmonella* species [57].

#### 1.3.2. Organic Acids

LAB are well-recognized for their pivotal role in fermentation, where they produce various organic acids that are essential metabolic products [29]. The combined effect of these organic acids, such as lactic and acetic acids, not only shapes the flavor, texture, and shelf life of fermented foods but also contributes to food safety by inhibiting the growth of spoilage organisms and pathogens [3]. The antimicrobial effects of these acids are primarily due to their action on the bacterial cytoplasmic membrane, disrupting the membrane potential and hindering active transport mechanisms, ultimately leading to the inhibition of harmful bacteria [58].

Lactic acid is the predominant organic acid produced by LAB and plays a central role in the fermentation of foods like yogurt, cheese, pickles, and sauerkraut [59]. Its production results from the metabolic breakdown of sugars, such as lactose in milk, by LAB species of *Lactobacillus* and *Streptococcus* [3]. The generation of lactic acid contributes significantly to the sour taste that characterizes many fermented foods, and it lowers the pH of the product, inhibiting the growth of pathogens such as *L. monocytogenes* and *E. coli* that do not thrive in the modified low-pH environment [44,60]. In yogurt production, for example, the co-fermentation of *S. thermophilus* and *Lactobacillus bulgaricus* leads to the formation of lactic acid, which thickens the milk proteins and gives yogurt its distinctive tangy flavor and creamy texture. In cheese-making, lactic acid promotes the coagulation of casein micelles, leading to curd formation and contributing to the texture of different cheese varieties [61,62].

Acetic acid is a significant metabolite produced by acetic acid bacteria but can also be produced by LAB through the heterofermentative pathway of glucose metabolism, particularly in species such as *L. brevis* and *L. fermentum* [44,63]. Acetic acid has a higher pKa (4.75) than lactic acid (3.86), giving it stronger antimicrobial properties in low pH environments, as a larger proportion remains undissociated and can penetrate microbial cell membranes [64]. It, therefore, exhibits strong bacteriostatic activity, effectively inhibiting the growth of a broad range of harmful microorganisms, including yeasts, fungi, and various bacteria. Its antimicrobial mechanism involves several pathways, including interference with the synthesis of key bacterial enzymes, the reduction in pH, which creates an unfavorable environment for pathogens, and the disruption of bacterial cell membranes, especially in Gram-negative bacteria [44]. Its sharp, vinegary taste, combined with its potent antimicrobial effects, makes acetic acid particularly effective in preserving foods such as pickles and vinegar [65].

#### 1.3.3. Gamma-Aminobutyric Acid (GABA)

GABA is a neuroinhibitory amino acid that is naturally present in mammals and plants, although its levels are typically low, leading to the need for chemical synthesis or bioconversion using microorganisms [44]. However, since chemically synthesized GABA is prohibited in the food industry, bioconversion using food-grade LAB has become a key method for producing GABA or foods rich in GABA [66]. GABA is produced by LAB through the decarboxylation of L-glutamate by the enzyme glutamate decarboxylase (GAD) under anaerobic conditions, accompanied by proton consumption [42]. The GadC antiporter facilitates the transport of L-glutamate into the cell, where it is decarboxylated by GAD, with pyridoxal-5′-phosphate (PLP) as a cofactor, producing GABA that is exported from the cell back into the extracellular matrix and releasing CO_2_ [67].

Various GABA-producing LAB strains of *Lactiplantibacillus paraplantarum* and *Levilactobacillus brevis* have been isolated from fermented foods like cheese, kimchi, yogurt, and soy-based products, with ongoing optimization efforts [42,43]. Different studies have shown that GABA exhibits a range of physiological benefits, including antidepressant, antianxiety, and antihypertensive effects [68,69]. GABA-rich foods produced by LAB have also been reported to deliver similar therapeutic benefits in the reduction in depression [70]. Due to its ability to reduce neuronal activity and prevent nerve cells from overheating, GABA is widely used in treating psychiatric disorders like epilepsy, convulsions, and Parkinson’s disease, highlighting its potential in both food and medical applications [71].

### 1.4. The Challenge/Burden of Foodborne Illnesses

Foodborne illnesses have become a significant global health threat, affecting over 600 million people annually [5]. These diseases are caused by a wide range of pathogenic microorganisms such as *Salmonella*, *Campylobacter*, and *E. coli* that, at one stage or another, contaminate food during production, processing, storage, and distribution, threatening not only public health but also the global economy [6,7]. Currently, about 75% of newly discovered and emerging diseases, including foodborne pathogens, originate from animals, accounting for most zoonotic infections in humans [72]. These pathogens tend to proliferate along the farm-to-table chain, resulting in large-scale outbreaks that affect millions of people every year [5]. The persistence of these pathogens in the food chain can be attributed to their transmission within food animals and the agricultural environment via a variety of pathways, including contaminated feed, water, humans, flies, rodents, and other vectors [72].

The burden of these foodborne illnesses in both developing and developed countries remains high and results in several outbreaks, causing people to be hospitalized, boosting pressure on economic systems, long-term health effects, and even death under severe conditions [73]. For instance, in the African region where the burden of foodborne illnesses is most severe, the 31 foodborne hazards identified by the WHO in 2010 resulted in an estimated 1200–1300 disability-adjusted life years (DALYs) per 100,000 people, compared to 35–711 in other regions of the world, of which almost 70% was caused by diarrheal disease agents, primarily non-typhoidal *Salmonella*, Enteropathogenic and Enterotoxigenic *E. coli*, *Vibrio cholerae*, etc. [74]. In 2011, studies for the CDC estimated that the United States experienced approximately 48 million new cases of foodborne illnesses annually, leading to 128,000 hospitalizations and 3000 deaths [75]. In the United Kingdom and Wales, about a million cases of foodborne illness were estimated in 2011, with about 20,000 hospitalizations and 500 deaths annually [76]. According to the WHO, children below the age of five are most vulnerable, making up nearly 30% of deaths due to foodborne disease illnesses [77]. This is attributed to the developmental stages in their system and poor immunity to several infections. Besides the health effects, the economic toll caused by foodborne diseases would be thousands of medical costs, lost productivity, and disrupted functioning of supply chains related to food [7].

### 1.5. Safety of Fermented Foods and Beverages

Fermented foods and beverages, produced for centuries through natural fermentation by indigenous microorganisms in raw materials or the environment, offer unique flavors, enhanced nutritional benefits, and extended shelf life [8]. From yogurt and kimchi to kombucha and kefir, these foods are an integral part of numerous cultures because they contribute to health and the economy [2]. However, like other food products, fermented foods and beverages have safety concerns that require a proper understanding of the process of fermentation involved and the microbial activity occurring to avoid health risks. Safety in fermented foods depends largely on maintaining the right balance of beneficial microorganisms during the fermentation process [8]. If the environment for fermentation is not optimal—such as due to poor hygiene or failure in temperature control—the beneficial microbes may not be dominant, and contamination with harmful bacteria, molds, or toxins can occur [9]. While fermented foods are generally considered safe, they involve some degree of risk, which might come from poor control of proper fermentation practices. Poorly controlled fermentation conditions allow the proliferation of pathogenic bacteria such as *Salmonella*, *E. coli*, and *L. monocytogenes* [72]. In particular, inadequate fermentation, which allows for insufficient acidity or anaerobic conditions, can also permit the growth of spoilage bacteria or even toxin-producing pathogens like *Clostridium botulinum* [8].

Commercial production of fermented foods usually involves the use of LAB starter cultures that ensure consistent and safe fermentation. These selected strains enhance flavor and texture while promoting food safety through the out-competition of harmful microorganisms [3]. However, the risk of *E. coli* and *Salmonella* contamination in fermented foods increases in certain rural areas where access to clean water is limited and potentially contaminated water sources like streams or rivers are used in production [9]. Hence, quality control measures in commercial operations, including regular monitoring of pH levels and microbial counts, can help ensure the fermentation process’s safety and the absence of contaminants. Also, biogenic amines are one of the potential risks of fermented foods, and their formation occurs during fermentation by naturally occurring or starter culture microorganisms [78]. The production of biogenic amine in fermented foods is influenced by raw material composition, fermentation time, and conditions of processing and is capable of eliciting allergic symptoms among sensitive individuals [8]. Generally, high accumulation of biogenic amines (BA) in products is related to spoilage, and though certain strains of LAB from species such as *L. plantarum* or processes can degrade these compounds [79,80], good control of raw material quality and optimization of fermentation conditions remain the best strategy and should be highly considered in the production of fermented foods.

## 2. Role of LAB in Food Safety

LAB play a very important role in food safety through their activities as natural biopreservatives in many fermented foods and beverages [59]. These microorganisms are usually present in whole dairy products, meats, and most plant-derived foods, where they improve the safety of foods by means of organic acid production, bacteriocins, and other substances with antimicrobial properties [44]. Through the production of metabolic byproducts of fermentation, such as lactic acid, LAB lowers the pH of the food products and creates an acidic environment that is unfavorable for many spoilage microorganisms and pathogens such as *Salmonella*, *Listeria*, and *E. coli* [58].

### 2.1. Inhibition of Pathogens

As previously stated, LAB are generally known for their antimicrobial properties, which aid in preventing and reducing foodborne illnesses through the inhibition of pathogenic microbes in food products [43]. They significantly contribute to food safety through the production of different substances such as organic acids, bacteriocins, hydrogen peroxide, and enzymes, all of which act to suppress the growth of noxious microorganisms [55,58]. During the fermentation process, LAB produce metabolites such as bacteriocins, which interfere with microbial cell membranes, inhibit the synthesis of enzymes involved in cell wall construction, interfere with the cellular proton gradient, and induce the production of reactive oxygen species, which ultimately increases the oxidative stress within the pathogen cells leading to their death [60]. Most scientific reports have come to indicate that LAB usually inhibits pathogenic microorganisms by providing conditions unfavorable for the growth of the pathogens through a significant reduction in pH, caused mainly by the production of lactic acid [3,59,81]. This low pH acts to impede the growth of bacteria, including *Salmonella* spp., for which optimal growth occurs within a pH range of 4.0 to 9.0 and is sensitive to more acidic conditions [82]. Other organic acids, such as acetic and propionic acids, which are produced as end products of fermentation, still show antagonistic effects against bacteria, yeasts, and fungi even though they are produced in smaller amounts [60]. LAB also contribute to the inhibition of pathogens in fermented foods by competitive exclusion [83]. In the process of fermentation, LAB quickly become the dominating microbial flora by competing with potential pathogens for nutrients and space. With their fast multiplication and production of antimicrobial compounds, they prevent the establishment of harmful microorganisms in the food matrix, thereby reducing foodborne illness [84].

### 2.2. Biopreservation

Biopreservation is a food preservation technique that involves inoculating food with nonpathogenic microorganisms, known as protective cultures, to inhibit the growth of harmful or undesirable microbes [85]. With the increase in demand for healthier and more natural food products, interest in the use of biopreservation techniques to extend shelf life and improve food safety has also been on the rise [86]. The common approach to biopreservation includes the application of antimicrobial compounds that selectively target foodborne pathogens and spoilage bacteria without affecting the intestinal microbiome of the consumer, implying that a biopreservative agent should be selective in action [87]. The agents can also be part of a hurdles technology approach combining several preservation methods for synergistic effects, thus perhaps allowing lower doses of preservatives or milder technological treatments [88]. In this respect, LAB plays a very important role in biopreservation by providing an alternative to synthetic chemical preservatives that are both effective and of natural origin. Thus, it is in the mode of their antimicrobial properties that LAB can impede the growth of pathogenic microorganisms, extending the shelf life of food products and enhancing their safety without the need for chemical preservatives [89]. Additionally, LAB fermentations improve the nutritional quality of the final product by enhancing the bioavailability of certain nutrients, such as amino acids, and promoting the synthesis of compounds with beneficial activity for the host, such as vitamins and bioactive peptides [90]. In this way, LAB strains applied to biopreservation enhance not only the safety but also the health-promoting properties of foods, adding to their value as natural preservatives. In their study to evaluate the effectiveness of LAB in preserving fresh beef at room temperature, [91] applied two LAB strains, *Pediococcus pentosaceus* LIV 01 and *P. acidilactici* FLE 01, to beef samples and incubated them at 30 °C for 7 days. The results showed that the presence of pathogenic bacteria, including *Enterobacteriaceae*, *Staphylococcus*, and coliforms, was reduced and that thiobarbituric acid and free fatty acid levels were lower in LAB-inoculated samples compared to the controls, demonstrating the potential of LAB as biological preservatives for improving safety and shelf life of fresh beef.

### 2.3. Reduction in Toxins

Biogenic amines (BAs) are non-volatile, low-molecular-weight nitrogenous compounds produced through the decarboxylation of amino acids, usually as part of normal metabolic processes in humans, animals, plants, and microorganisms [92]. Amino acid decarboxylases, the enzymes responsible for this process, are commonly found in spoilage microbes and food microorganisms, including naturally occurring or added LAB involved in food and beverage fermentation, although this occurs in very low quantities [78]. The large presence of BAs in food, especially histamine and tyramine, deteriorates the nutritional and sensory qualities of the products and can cause food intoxication when consumed, with symptoms manifesting as allergic reactions, headache, diarrhea, vomiting, and abdominal pain [80]. LAB has been shown to play an important role in inhibiting bacterial growth and decreasing the harmful BA formation produced by microorganisms through amino acid decarboxylation during food fermentation or spoilage.

Though certain LAB strains of *L. buchneri*, *L. parabuchneri* and *L. rhamnosus* have been shown to possess decarboxylase enzymes responsible for the conversion of amino acids into BAs, thereby contributing to BA accumulation [80,93,94], strains of *L. plantarum* have been shown to exhibit amine oxidase activity or produce metabolites that degrade or inhibit BAs, indicating that the presence of the decarboxylase enzymes in LAB is basically strain-dependent [78,95]. The study by [79] investigated the impact of autochthonous starter cultures (*L. plantarum*, *L. salivarius*, and their combination) on the accumulation of BAs in traditional Chinese smoked horsemeat sausage. They found that the starter cultures effectively inhibited the growth of indigenous bacteria and promoted BA degradation through oxidative deamination by amine oxidase. Among them, strains of *L. salivarius* showed the greatest ability to reduce BAs, while the combination of *L. plantarum* and *L. salivarius* had a synergistic effect. Similarly, Lee et al. [96] assessed 1448 LAB strains isolated from various kimchi varieties and found that five strains significantly degraded histamine and/or tyramine. These strains included *Levilactobacillus brevis* PK08, *Lactiplantibacillus pentosus* PK05, *Leuconostoc mesenteroides* YM20, *L. plantarum* KD15, and *Latilactobacillus sakei* YM21.

Mycotoxins, on the other hand, are toxic secondary metabolic products of molds that include, among others, *Aspergillus*, *Fusarium*, and *Penicillium*, which poses a significant food safety issue due to mold growth during the production, storage, or processing of fermented foods [97]. There are an estimated 300 to 20,000 different types of mycotoxins in nature produced by different molds, with aflatoxins, deoxynivalenol, fumonisins, ochratoxins, and zearalenone being the most common contaminants globally [98]. Chronic exposure to mycotoxins above established limits in food can result in serious health issues, such as immune suppression, carcinogenicity, mutagenicity, organ toxicity, and birth defects [97,98]. LAB has been reported to significantly contribute to the detoxification of these mycotoxins in fermented foods and beverages by hydrolyzing them to less or non-toxic forms [99]. The metabolites produced by LAB, such as organic acids, phenolic compounds, fatty acids, and bioactive peptides, can interact and bind to mycotoxins in fermented foods to reduce their toxicity [100]. In a study by Gerbaldo et al. [101] to demonstrate the antifungal and mycotoxin-reducing abilities of LAB, *L. rhamnosus* L60 and *L. fermentum* L23 produced secondary metabolites, including organic acids, bacteriocins, and hydrogen peroxide (in the case of L60), and these were effective in inactivating *Aspergillus* section *Flavi*, known for producing the harmful mycotoxin aflatoxin B1. Through both qualitative and quantitative methods, it was found that both L60 and L23 completely inhibited the growth of aflatoxin-producing fungal strains. Additionally, *L. rhamnosus* L60 reduced aflatoxin B1 production by 95.7–99.8%, while *L. fermentum* L23 reduced it by 27.5–100%. The detoxification mechanisms associated with LAB in fermented food products could occur through the production of diverse proteolytic enzymes. These enzymes are capable of hydrolyzing proteins, including cell-wall bound proteinase, which can hydrolyze proteins into polypeptides and abundant intracellular peptidases that degrade the transferred peptides to amino acids [100,102]. Additionally, mycotoxins can be detoxified through adsorption by the cell wall of LAB, which are facilitated by the structural components present in the bacteria, such as peptidoglycan, polysaccharides, and proteins [100,103].

## 3. Enhancing Food Quality with LAB

LAB are of great importance to the fermentation process due to their contribution through a unique enzymatic system that produces extra-cellular protease and extra-cellular lipase enzymes that hydrolyze proteins, fats, and carbohydrates into smaller molecules [10]. The foregoing enzymatic activity of LAB encourages complexity in flavor and improves digestibility, hence increasing nutritional value. LAB are at the center of fermentation in different food products driven by dominant species such as *L. plantarum*, *L. sakei*, *L. paracasei*, and *L. fermentum* [104]. The improvement in the quality of food brought about by LAB is a result of organic acid production, primarily lactic acid, through fermentation, which lowers pH and renders the environment unsuitable for the growth of undesirable bacteria, thus enhancing shelf life and food safety [3,59]. Additionally, the proteolytic and lipolytic activities of LAB are involved in protein and fat degradation, releasing peptides, amino acids, and fatty acids, respectively, all important compounds for flavor development [104]. For instance, in fermented meat products, the action of these enzymes provides a tender and flavorful product. On the contrary, the degradation of proteins will not only improve the texture but also form bioactive peptides possessing antihypertensive, antioxidant, and immune-modulating activities [105,106]. As a result, this enhances the sensory qualities, nutritional profile, and safety of food, generally improving the quality of fermented foods and beverages. Their application in traditional and industrial processes of fermentation is, therefore, a cornerstone for current modern food biotechnology.

### 3.1. Flavor Development

**Figure 2:**
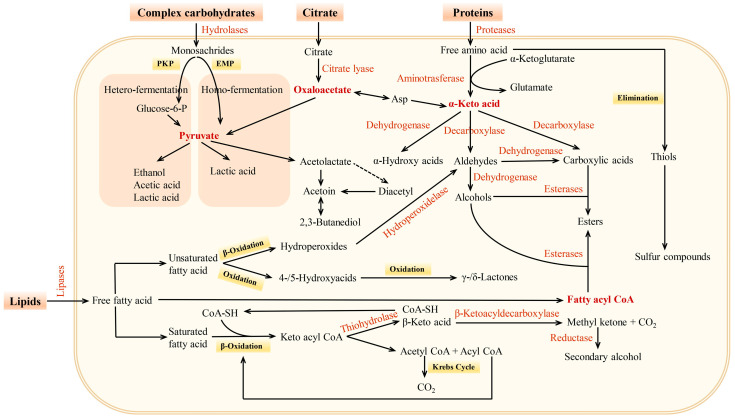
Overview of general metabolic pathways used by LAB for flavor formation in fermented foods [11].

LAB form a very important group in the process of fermentation and highly contribute to the development of flavors, aromas, and textures within a wide range of fermented foods and beverages [36]. These microorganisms, which include species of *Lactobacillus*, *Lacticaseibacillus*, *Limosilactobacillus*, *Leuconostoc*, and *Pediococcus*, produce enzymes during fermentation that can hydrolyze numerous food components like carbohydrates and lipids into flavor precursors that can be converted into aroma compounds with complex sensory profiles [11,12]. Acidification (carbohydrate fermentation), fatty acid metabolism, and amino acid catabolism are examples of some metabolic processes that LAB utilize during fermentation to contribute to the development of the organoleptic properties of fermented products [107], including those characteristics that give fermented foods their unique flavors, aromas, and textures, such as in yogurt, cheese, sourdough, and kimchi [2]. A general overview of the metabolic pathways used by LAB for flavor formation in fermented foods is presented in Figure 2.

From an organoleptic perspective, the sourness from lactic acid balances other flavor elements in many LAB-fermented foods and brings a refreshing tartness to products such as yogurt and sauerkraut [36]. In sourdough, for instance, where the proteolytic activity of LAB is relatively limited, the process of acidification resulting from the breakdown of carbohydrates triggers the activation of endogenous cereal proteases [46]. These enzymes, naturally present in the grains, are stimulated by the lowered pH during fermentation. As a result, they break down proteins in the flour, releasing peptides and amino acids. These smaller molecules can then be utilized by LAB as essential building blocks and energy sources for their metabolic processes to produce a range of flavor-active compounds that directly impact the flavor, aroma, and overall sensory profile of sourdough [108]. For instance, the conversion of arginine to ornithine via the arginine deiminase (ADI) pathway, particularly in species like *L. reuteri*, *L. pontis*, *L. fermentum*, *L. brevis*, and *L. sakei*, plays a crucial role during sourdough fermentation. The ADI pathway not only increases the competitiveness of these LAB strains but also produces ornithine, a precursor to 2-acetyl-1-pyrroline, which is responsible for the characteristic aroma of baked wheat bread crust [46].

The breakdown of lipids and the oxidation of fatty acids in fermented foods are key contributors to the formation of flavor compounds. Lipid hydrolysis, which involves the breakdown of triglycerides, diglycerides, and monoglycerides, releases free fatty acids that serve as essential aroma compounds in many fermented food products [109]. LAB species of *Lactococcus*, *Lactobacillus Lacticaseibacillus*, and *Limosilactobacillus* are known to produce lipases, enzymes that facilitate this process by generating free fatty acids, which play a crucial role in the development of aroma in a wide range of fermented foods [11]. The oxidation of lipids in fermented foods leads to the formation of various volatile compounds such as esters, lactones, alkanes, secondary alcohols, and methyl ketones [110]. Saturated fatty acids can undergo *β*-oxidation, producing odd-carbon methyl ketones, which are then converted into secondary alcohols by reductases [111]. For unsaturated fatty acids, their oxidation mainly occurs through two pathways: the free radical oxidation pathway, which involves the formation of hydroperoxides via free radicals, and the other hydroxy acid formation pathway, which produces hydroxy acids that are further converted into lactones, which contribute strong fruity flavors in fermented foods [11]. Additionally, LAB produces esterases that synthesize flavor esters in fermented foods, like ethyl butanoate and ethyl hexanoate, through reactions between alcohols and acids, which provide fruity and floral notes that are especially relevant in fermented beverages, such as wine and beer [11,112].

### 3.2. Textural Improvements

LAB contribute to the textural development of many fermented products, such as yogurt, cheese, sourdough bread, and pickled vegetables, through their metabolic activities such as acidification, exopolysaccharide (EPS) production, and enzymatic reactions [11,13]. These characteristics not only enhance the nutritional profile of fermented foods but also contribute to their sensory and organoleptic qualities. Acidification, in the case of dairy products like yogurt and cheese, causes coagulation of the milk proteins, most of all casein, to form a gel-like structure [113]. The final product texture will range from very soft to very firm, smooth, or with mouthfeel, depending on the degree of acidification and speed of acidification. In yogurt, for example, there is a possibility of developing a higher acidity and, hence, a firmer texture or one with slower fermentation, leading to creamier and softer consistencies [113]. LAB is very important in sourdough bread in terms of gluten modification and dough structure. Acidification from LAB during fermentation strengthens the gluten network, thereby improving the elasticity and extensibility of dough [114]. These structural changes in both the crumb and dough are largely attributed to the proteolysis of gluten proteins, primarily driven by the increased acidity from the organic acids produced during sourdough fermentation, which also helps to control the rate of fermentation and give the dough enough time to develop in texture and flavor [115,116].

Due to their wide applicability in the food industry, special attention has been directed toward EPS produced by LAB. These biopolymers can form a loosely bound layer or be secreted into the extracellular space, significantly impacting the texture and structure of fermented foods [13]. LAB strains of genera such as *Lactobacillus*, *Lactococcus*, *Fructilactobacillus*, *Lacticaseibacillus*, and *Lactiplantibacillus* have gained particular focus, especially in dairy products where starter strains capable of producing EPS are sought for their beneficial effects on food quality [117]. EPS function as natural thickeners and texturizers by increasing viscosity and act as stabilizers by binding water and interacting with other milk constituents, such as proteins and micelles, to enhance the firmness of the casein network in dairy products like yogurt and cheese [13]. One of the most important contributions of EPSs is their ability to reduce syneresis (the separation of liquid from a gel-like substance), which is particularly advantageous in dairy food processing. This moisture retention ensures the product maintains a smooth and consistent texture, preventing the release of excess liquid, which can negatively affect the sensory experience [45]. This is particularly important in low-fat or fat-free products, whereby the lack of fat can otherwise compromise the desirable mouthfeel. LAB-derived EPSs are also used in the food industry for their emulsifying and gelling properties. These attributes influence the rheology, firmness, and overall sensory properties, resulting in enhanced mouthfeel and product stability [117].

### 3.3. Preservation of Color and Freshness

There are various traditional methods for food preservation, such as refrigeration, freezing, pasteurization, boiling, and sterilization, as well as the use of chemical preservatives like sodium chloride, sodium nitrite, and benzoates [118]. However, these methods may pose health risks, alter the sensory characteristics of food, and cause the loss of nutritional elements. As a result, biopreservation techniques, particularly those involving LAB and their metabolites, are becoming more favorable for improving food quality and safety since they offer the advantage of extending shelf life while maintaining the food’s hygiene, nutritional content, and sensory properties [14]. The acidic environment created by LAB hinders the fast proliferation of spoilage microorganisms and prevents changes in color that would usually develop from microbial actions or enzymatic reactions under a more alkaline environment during storage [3]. For example, in fermented meat products, such as sausages or cured meat products, LAB inhibit the browning or discoloration by spoilage bacteria through the production of organic acids, which stabilize the red color, thus keeping the product more appealing for a longer period [119].

In vegetable fermentation, such as in sauerkraut or kimchi, the biopreservative activities of LAB inhibit microbial and enzymatic actions/reactions that could otherwise lead to the degradation of vibrant colors in fresh produce, protecting food from oxidative processes, which are responsible for the fading or browning of color in many foods [120]. Oxidation, particularly in fruits, vegetables, and meat, can cause color changes and impact the overall quality of the product [121]. LAB’s metabolic activities, including the production of enzymes such as glutathione reductase, help limit oxidative reactions, thereby preserving the natural color of the food [120].

## 4. Nutritional Benefits of LAB in Foods and Beverages

LAB-fermented foods have been a staple in human diets since ancient times, initially prized for their extended shelf life and unique organoleptic qualities. Today, these foods are also consumed with a growing awareness of their health benefits [122]. During fermentation, LAB not only transform the flavor and texture of foods but also release a variety of bioactive compounds and signal molecules that can positively influence human health. These beneficial bacteria and their metabolites interact with the gut microbiome, functioning almost like an orchestra, working together to maintain intestinal and overall body health [15]. The nutritional benefits of LAB in fermented foods and beverages go far beyond flavor and preservation. These bacteria enhance the bioavailability of essential nutrients by breaking down complex compounds, making them easier for the body to absorb [17]. For example, in dairy products like yogurt and kefir, LAB improve protein digestibility and reduce lactose content, making these foods more suitable for lactose-intolerant individuals [123]. Moreover, LAB are capable of synthesizing important vitamins such as B12, riboflavin, and folate, contributing directly to nutritional intake [124].

In addition to improving nutrient content, LAB strains act as probiotics, promoting gut health by maintaining a balanced microbiome. Strains of *Lactobacillus acidophilus* and *Lactiplantibacillus plantarum* help prevent gastrointestinal issues and support immune function [18]. LAB also produce bioactive compounds like conjugated linoleic acid (CLA), known for its anti-inflammatory and anti-carcinogenic properties, further amplifying the health benefits of fermented foods [125]. They also contribute to cardiovascular health by lowering cholesterol levels through the assimilation of cholesterol and the binding of bile acids, promoting their excretion from the body [126]. In fermented beverages like kombucha and kefir, LAB provide hydration alongside a rich source of probiotics, antioxidants, and organic acids, which support digestion and detoxification processes [127]. These beverages are not only refreshing but also packed with health-promoting compounds. Through their metabolic actions, LAB play a vital role in maintaining the balance of our intestinal ecosystem, contributing to homeostasis and overall health.

### 4.1. Probiotic Properties

LAB, particularly strains from genera like *Lactobacillus* and *Lactiplantibacillus plantarum*, are widely recognized for their probiotic properties, offering a multitude of health benefits when consumed in fermented foods and beverages [18]. Probiotics, as defined by the Food and Agriculture Organization of the United Nations (FAO) and World Health Organization (WHO), are “live microorganisms which, when administered in adequate amounts, confer a health benefit on the host” [128]. LAB probiotics are known for their ability to survive the acidic environment of the stomach and colonize the intestinal tract, where they contribute to the balance of the gut microbiota [129]. This is crucial for digestive health, as a balanced gut microbiome is linked to improved digestion, nutrient absorption, and the prevention of gastrointestinal disorders such as irritable bowel syndrome (IBS), constipation, and diarrhea [130].

*Lactobacillus acidophilus*, *Lacticaseibacillus casei*, *Lactiplantibacillus plantarum*, and *Lacticaseibacillus rhamnosus* are among the most used probiotics in food products [18]. Lactobacilli have been intensively investigated and, relative to other bacterial genus’, are well described in terms of genomes and their interactions with humans in terms of both health and disease [131]. They have also been given the designation “qualified presumption of safety” (QPS) by the European Food Safety Authority (EFSA) and “generally recognized as safe” (GRAS) by the U.S. Food and Drug Administration (USFDA), which makes them excellent choices for use as probiotics in food manufacturing [131]. Lactobacilli are non-pathogenic and are known to produce many beneficial substances such as bacteriocins, hydrogen peroxide, and organic acids like lactic acid during fermentation, which not only helps preserve food but also lowers the pH in the gut, creating an environment that is unfavorable for pathogenic bacteria [3,132]. LAB modulate the host’s immune response by interacting with the gastrointestinal mucosa and influencing immune cells such as macrophages, dendritic cells, and regulatory T cells [133]. Their cell wall components, like lipoteichoic acids (LTA) and peptidoglycans, are recognized by pattern recognition receptors (e.g., toll-like receptors), triggering immune activation, though different LAB may elicit varied immune responses based on their unique surface molecules and secreted proteins [133,134]. In addition, they contribute to the breakdown of lactose, aiding in the digestion of dairy products, which is particularly beneficial for individuals who are lactose intolerant [135]. The study by Gingold-Belfer et al. [136] evaluated the effectiveness of probiotics with β-galactosidase activity in alleviating symptoms of lactose malabsorption and improving the results of the lactose hydrogen breath test (LHBT). In the study, eight symptomatic female patients with positive LHBT results were treated for six months using a probiotic formula known as Bio-25, which provides 25 billion active bacteria per capsule and contained strains of LAB from species such as *L. acidophilus*, *L. rhamnosus*, *L. casei*, and *S. thermophilus*. The results showed that Bio-25 effectively reduced the frequency and severity of lactose intolerance symptoms, particularly bloating and flatulence, indicating its potential as a treatment for lactose malabsorption.

### 4.2. Enhanced Bioavailability of Nutrients

Good health largely depends on proper food intake that meets the body’s nutritional needs, as nutrients like minerals are essential for various vital functions, including bone development and nerve transmission [17]. Poor nutrition results in insufficient necessary trace nutrients in the form of zinc, iron, and calcium, leading to malnutrition, anemia, risk of infections, and osteoporosis [137]. Although many essential nutrients, such as vitamins and minerals, are present in a wide variety of foods, their bioavailability, or the extent to which the body can absorb and utilize them, is often limited. Factors like the presence of antinutritional compounds (e.g., phytates and oxalates) in plant-based foods can bind to these nutrients, reducing their absorption in the body [16]. As a result, despite consuming nutrient-rich foods, populations, particularly in developing countries, may still suffer from widespread deficiencies in key nutrients like zinc, iron, and calcium [138]. Fermentation, or more importantly, LAB fermentation, has been known to degrade anti-nutritional factors within foods and add further accessibility to these nutrients for absorption. [17] As a result, LAB fermentation significantly enhances the bioavailability of vitamins, minerals, and other bioactive compounds, improving overall nutrient intake and promoting better health outcomes. The study by Rekha and Vijayalakshmi [139] demonstrated the potential of LAB to improve nutrient content in fermented foods. In the study, five probiotic LAB strains (*L. acidophilus*, *L. bulgaricus*, *L. casei*, *L. plantarum*, and *L. fermentum*), along with *Saccharomyces boulardii* yeast, were used to ferment soymilk, significantly increasing the bioactive isoflavones genistein and daidzein. High-performance liquid chromatography showed a 97–98% increase in genistein and a 62–92% increase in daidzein after 24 to 48 h of fermentation. The study also found that the fermentation enhanced the bioavailability of minerals and B vitamins, reduced antinutrients like phytic acid, and supported LAB viability.

Fermentation by LAB also improves the bioavailability of bioactive compounds, including antioxidants and polyphenols, which have health-promoting properties. Phenolic compounds naturally exist in vegetables and fruits, but the complex molecular structure of phenolics may reduce their bioavailability within the human body [140]. LAB help increase the bioavailability of such compounds through degradation mediated by enzymatic activities in decarboxylation, deglycosylation, or de-esterification processes [66]. LAB break down the polyphenols during fermentation into simpler forms, such as aglycones, which are then more easily absorbed in the intestine. This microbial transformation increases the health value of polyphenols, improving their antioxidant and other protective activities in fermented foods [140]. In this way, LAB releases these bioactive compounds from the food matrix into a more available form, rendering them more useful for human health.

### 4.3. Synthesis of Functional Compounds

LAB have widely been studied for their role in food and beverage fermentation. Their ability to synthesize functional compounds with immense health benefits makes them important in enhancing the nutritional value of foods. The functional compounds synthesized by LAB include some essential vitamins, such as folate, riboflavin (B2), and B12, and short-chain fatty acids (SCFAs), which play important functions like promoting gut health and improving the general nutritional profile of fermented products [19]. However, it is noteworthy that the capability of LAB in the production of these vitamins is usually dependent on the species and strain. For instance, LAB strains such as *S. thermophilus* and *L. lactis* are mostly used in yogurt production as starter cultures due to their ability to synthesize folate [141]. Folate is an important vitamin that is highly required for DNA synthesis, cell division, and the proper development of neural tissues [142]. Its deficiency is associated with a number of health disorders, including increased oxidative damage and altered levels of base excision repair, which are associated with neurological disorders [143]. During food fermentation, LAB can increase levels of folate by converting precursors of folic acid into bioavailable forms, thus improving their nutritional values and making fermented dairy products such as yogurt and cheese good contributors to the intake of folate by humans [19].

Vitamin B12 is highly crucial in nerve functioning, the production of red blood cells, and DNA synthesis [144]. Compared to non-vegetarians, vegetarians have lower vitamin B12 levels and are at a higher risk of deficiency, as vitamin B12 is naturally produced only by microorganisms and is primarily found in animal-based foods, making it challenging for those on vegetarian or vegan diets to meet their B12 requirements [145]. Certain LAB strains of *L. reuteri* have been shown to synthesize vitamin B12 during the fermentation of foods, providing an alternative source of this vital nutrient [124,146]. Production of B12 by LAB in fermented plant-based foods is, therefore, an accessible alternative for those who do not have much of an intake of food that comes from animal sources, increasing nutritional variety in their diets. Besides their role in the synthesis of vitamins, LAB also play a very important role in the production of SCFAs (mainly acetate, propionate, and butyrate) during fermentation of dietary fibers in the gut [147]. These are important metabolic substrates for colonocytes and participate in gut health maintenance, regulation of inflammation, and modulation of immune responses [148]. SCFAs can act as histone deacetylase inhibitors (HDACIs), influencing epigenetic regulation of gene expression [149]. By inhibiting histone deacetylases, SCFAs can modify chromatin structure, leading to changes in the expression of genes involved in key cellular processes like survival, proliferation, and differentiation [150]. In various in vitro cancer studies [151,152,153], SCFAs have been shown to exert anti-cancer effects, such as inhibiting the growth of breast, gastric, and cervical cancer cells, causing cell cycle arrest, triggering apoptosis through mitochondrial pathways, and promoting autophagy. These effects are often accompanied by increased production of reactive oxygen species, further contributing to cancer cell death [150]. As a result, SCFAs are recognized for their potential therapeutic role in cancer treatment.

### 4.4. Reduction in Anti-Nutritional Factors

Various anti-nutritional factors with toxic potential have been identified in food, including lectins, tannins, saponins, amylase inhibitors, phytic acid, gossypol, protease inhibitors, metal-binding compounds, antivitamin factors, and goitrogens [138]. These compounds, often found in the seeds of cereals and legumes, can interfere with nutrient absorption and lead to health issues such as poor digestion and nutrient deficiencies [17]. Some of these anti-nutrients are heat-labile, meaning they can be reduced through cooking, while others are heat-stable and more difficult to eliminate, posing challenges in improving the nutritional value of certain plant-based foods, particularly in regions where these crops form dietary staples [138]. LAB fermentation has proven to be an effective method for reducing these anti-nutritional factors and a better alternative for minimizing their adverse effects in diets [139]. During fermentation, LAB produces enzymes like phytase, which degrades the phytic acid into inositol and free phosphate. This process decreases phytic acid levels in food, releasing minerals like calcium and iron that are typically bound to it, thereby improving their bioavailability [154]. Similarly, the study by Nivetha et al. [155] found that LAB fermentation of linseed beverage using *L. acidophilus* alongside yeasts and other probiotic strains led to a 58% reduction in total phenolics, 66% reduction in tannins, and a 65% reduction in cyanogenic glycosides compared to raw linseed. The study attributed the reduction in these antinutritional factors to the production of enzymes like linamarase and β-glucosidase by LAB during the fermentation process. Ogodo et al. [156] examined how fermentation with a LAB consortium affects anti-nutritional factors in maize flour and found that increased fermentation periods led to a significant (*p* < 0.05) reduction in the antinutritional factors, including tannins, phytate, polyphenols, and trypsin inhibitor activity. They concluded that there was a more significant reduction in these anti-nutritional factors with LAB-consortium fermentation when compared with spontaneous fermentation.

## 5. Applications in Fermented Foods

LAB are an essential component of the traditional food fermentation process, which has been utilized for generations as a natural means of preserving and enhancing food [2]. Fermentation is still a common practice today despite its ancient origins, especially among indigenous tribes in Africa and other developing nations [138]. These tribes make a wide range of fermented foods and drinks that are prized for their unique flavor, texture, and appearance using LAB and yeast. LAB fermentation has several advantages over unfermented food. For instance, LAB create organic acids and antibacterial substances that enhance the flavor and safety of food while also preserving it [3]. A vast variety of fermented foods, such as cereal-based porridges, fermented fruits and vegetables, dairy products, and fermented meats, are produced in African societies, and LAB are essential to this process [2]. Though other types of fermentation, such as alkaline and amino acid fermentation, are also utilized, lactic acid fermentation is one of the most often used techniques in food processing, along with alcoholic fermentation.

During the fermentation process—for example, meats and fish, such as salami, sausages, or fish sauces—LAB operate on both texture and preservation, impeding spoilage and improving the sensory quality in general [13]. Even in cereal-based fermentations like in sourdough bread, LAB improve flavor, textural quality, and nutritional quality by hydrolyzing anti-nutritional factors and improving nutrient bioavailability [2]. These diverse applications show how versatile LAB could be in traditional and modern fermented foods, which are indispensable to guarantee safety, increase taste, and improve nutritional value.

### 5.1. Dairy Products

Over the past few decades, extensive research has shown the health benefits of fermented dairy products such as yogurt, cheese, kefir, and their traditional varieties, including improved digestibility, enhanced nutrition, and properties like anti-hypertensive and immune-boosting effects, leading to a growing consumer demand [157]. LAB are essential microorganisms in the production and preservation of these dairy-based fermented products as they play a central role in ensuring safety, enhancing flavor, and improving texture. Commonly used as starter cultures, LAB are introduced into raw materials, such as milk, with the purpose of accelerating and guiding the fermentation process [158]. Yogurt, for instance, is traditionally made by acidifying milk, a process that leads to the formation of curds (solidified portions of milk proteins) [59]. The acidification process is an essential process in the yogurt production process and is often intended to be quick and effective. It usually occurs as a result of the proliferation and activity of LAB, which leads to lactose fermentation [59]. Although two LAB species that are majorly involved in this process, *Streptococcus thermophilus* and *Lactobacillus delbrueckii* subsp. *bulgaricus* can independently grow in milk; they engage in a mutually beneficial relationship known as protocooperation [159]. Their interaction enhances yogurt production by improving texture through EPS formation, speeding up acidification, and enhancing flavor through the production of aromatic compounds [59,159].

Similar to yogurt production, LAB serve as essential starter cultures in cheese production and play a central role in the fermentation process, converting lactose into lactic acid, which lowers the pH of the product and creates an environment that is inhospitable for many pathogenic bacteria, and also leads to the curdling of milk which is a key step in forming cheese [160]. LAB also contribute to the biochemical transformations that occur during the ripening stage, which influence the texture, flavor, and overall quality of the cheese [161]. Historically, cheese was produced through spontaneous fermentation, relying on the natural microorganisms found in raw milk and its environment [160]. However, modern cheese-making involves LAB fermentation using strains such as *L. helveticus*, *L. lactis*, *L. delbrueckii*, and *S. thermophilus*, which are carefully selected to ensure consistency and enhance the desired characteristics of the final product [162]. These species are vital not only for fermentation but also for improving the sensory attributes and shelf life of cheese.

### 5.2. Vegetable Fermentation

LAB are fundamental to the fermentation of vegetables such as sauerkraut, kimchi, and pickles, where they significantly influence both safety and flavor. These microorganisms initiate and drive the fermentation process because they convert the available carbohydrates into lactic acid, which serves as a natural preservative, lowering the pH of the environment and contributing to the safety of fermented vegetables by inhibiting the growth of harmful bacteria and spoilage organisms, as well as preserving the vegetables for a long term [163]. The production of lactic acid from LAB during vegetable fermentation also ensures the production of a stable and safe product without chemical preservatives, therefore making fermented vegetables a natural and healthy food. Aside from their contribution to safety, LAB are important for determining the characteristic flavor profiles that fermented vegetables attain [29]. In metabolic activity, LAB degrade sugars and proteins into metabolites such as SCFAs, organic acids, EPS, and free amino acids. These metabolites bring about the usual tangy, sour, and complex flavors common in fermented products such as sauerkraut and kimchi [164]. LAB such as *L. plantarum* and *L. brevis* ferment sugars during vegetable fermentation into lactic acid and acetic acid, forming volatile compounds responsible for aroma and taste [163]. Besides enhancing flavor, these metabolites develop better texture, hence increasing the palatability of fermented vegetables.

LAB is involved in every stage of vegetable fermentation, and they play very important roles. Vegetable fermentation usually begins with hetero-lactic fermentation, which is dominated by LAB such as *L. brevis* that lower the pH, creating an environment less favorable for other microbes [165]. In the middle stage, homo-lactic fermentation takes over, where LAB such as *L. delbrueckii*, *L. plantarum*, and *Streptococcus* spp. convert over 80% of glucose into lactic acid [163]. As fermentation progresses, LAB produces organic acids, acetoin, ethanol, and amino acids, which contribute to the flavor and texture of the fermented vegetables. In the later stages, yeasts and acetic acid bacteria dominate, producing ethanol, acetic acid, and esters, which further enhance flavor complexity in fermented vegetable products [163].

Fermented chili peppers, for instance, have been found to be a prominent feature of local dishes in the southwestern region of China and in various cuisines worldwide [166]. Fermented chili pepper is majorly accepted by consumers due to its sensory qualities, which combine sour, spicy, umami, and salty tastes with its distinct scent [167]. LAB species such as *Lactobacillus versmoldensis* and *Levilactobacillus brevis* have been identified as major microorganisms highly involved in the spontaneous fermentation process that yield these sensory qualities [163]. In addition, fermentation of chili pepper leads to the production of various types of fermented products such as pickled chili pepper (*paojiao*), fermented chili pepper pastes, and fermented bean-chili paste (*doubanjiang*/*gochujang*) [166].

### 5.3. Meat and Fish Fermentation

LAB are commonly involved in fish and meat product fermentation and contribute enormously to their preservation, safety, and flavor. Fish, for instance, are highly susceptible to spoilage due to their neutral pH, high water content, and the presence of autolytic enzymes, which create favorable conditions for microbial growth and oxidative degradation [168]. While traditional preservation methods, such as heat treatment, can help reduce spoilage, they often compromise the fish’s nutritional quality and sensory characteristics, including flavor, appearance, and texture [169]. Chemical preservatives like butylhydroxyanisole and dibutylhydroxytoluene are also used but raise concerns about chemical residues [169]. Fermentation has, therefore, become a preferred preservation method over time, considering the several benefits it offers, including being cost-effective, environmentally friendly, and improving both the nutritional value and flavor of fermented fish [170]. During fish fermentation, LAB ferment the available sugars and carbohydrates, leading to the production of lactic acid that acts as both a natural preservative and for flavor improvement of the fish product. The fermentation by LAB and other microorganisms results in metabolic activities such as carbohydrate fermentation, protein degradation, and fat oxidation, giving a complex sensory experience with unique tastes, aromas, textures, and the overall quality of the fermented fish products [169]. While ancient fish fermentation was initially just considered a preservation method, over time, the focus has shifted to flavor optimization, and it is now beyond dispute that LAB plays an important role in the production of fermented fish with desirable sensory qualities [168]. For instance, the highly sought strong umami flavor in fermented fish, which is mainly brought about by glutamic acid and aspartic acid, is produced from the catabolism of proteins and nucleic acids into umami peptides, amino acids, and nucleotides due to the activities of LAB during fermentation [169]. The degradation of proteins into peptides and amino acids can be attributed to the strong proteolytic system in LAB, which provides substrates for microbial growth and multiplication and results in the production of metabolites that are associated with flavor.

LAB also perform several functions in meat fermentation. Besides the production of lactic acid, which increases the safety and acceptability of commodities such as salami and sausages, they also release enzymes such as protease and lipase [104]. These enzymes break down proteins, fats, and carbohydrates into smaller molecules, which in turn form flavorful compounds. Dominant LAB species involved in meat fermentation include *Lactiplantibacillus plantarum*, *Lacticaseibacillus paracasei*, *Limosilactobacillus fermentum*, among others [171]. Enzymatic activity from them causes hydrolysis of muscle proteins-myogenic fibrin and sarcoplasmic protein-into oligopeptides and amino acids, respectively. Such compounds undergo biochemical reactions to produce aromatic compounds responsible for enhanced flavor, including esters, alcohols, and SCFAs [104]. Lactic acid production by LAB affects the texture and safety of the meat product through its acidification, which reduces pH, inhibits spoilage bacterial growth, and thereby enhances the gel characteristic of the meats [3]. The synergy between LAB enzymes and those naturally present in meat induces biochemical changes that result in a more palatable product [104].

### 5.4. Cereal-Based Fermentation

Cereal fermentation has been an integral part of human food processing since ancient times, contributing significantly to the development of different foods such as sourdough bread. This fermentation process enhances the microbial stability of the final food product, creating a complex microbial ecosystem [172]. In fermented cereal-based foods, the activities of these microbes, particularly LAB, result in distinctive characteristics such as improved flavor, texture, and sensory appeal. Additionally, fermentation contributes to the stability, nutritional value, and potential health benefits of these products [173]. Traditional fermented foods made from common cereals like rice, wheat, maize, and sorghum are widely known across the globe and are valued for their enhanced palatability and overall quality [172]. The flavor profile of fermented cereal products greatly depends on the metabolic activities of LAB. LAB degrade complex carbohydrates, proteins, and lipids into a number of flavor-enhancing compounds such as organic acids, alcohols, esters, and aldehydes [174]. Lactic acid, which is the major product of the fermentation activity of LAB, determines the sour taste typical of fermented cereal products such as sourdough bread and fermented porridges [3]. LAB, particularly *Lactobacillus* spp., usually exhibits proteolytic activity, which is characterized by the degradation of proteins into peptides and free amino acids. Such proteolysis enhances the formation of bioactive peptides that are responsible for the overall sensory quality of fermented cereal products. Additionally, volatile compounds such as diacetyl and acetoin, produced through LAB fermentation, also contribute to buttery and fruity notes that improve the complexity of the flavor profile of fermented cereals [175].

Cereals generally contain little lipid, but fermented cereals have been shown to contain free fatty acids such as butanoic, hexanoic, and octanoic acids, which are released when LAB, particularly *Lactococcus* and *Lactobacillus* species, hydrolyze these lipids [11]. Through esterification with ethanol, LAB enzymes like esterases can transform these fatty acids into esters like ethyl butanoate and ethyl hexanoate [175]. In addition, these free fatty acids can undergo oxidation, resulting in the production of aldehydes, hydroperoxides, alkanes, methyl ketones, esters, and lactones, all of which are involved in the development of flavor in fermented cereals [11]. LAB also enhances the nutritional value of fermented cereals due to the breakdown of anti-nutritional factors, hence improving the bioavailability of vital nutrients [138]. Phytate, a common antinutritional factor found in cereals, binds essential minerals such as calcium, iron, and zinc, reducing their bioavailability, which can negatively affect the nutritional value of cereal-based foods [174]. LAB can express phytase activity at varying levels depending on the species and strain involved, which are particularly effective in degrading phytate, contributing to a significant reduction in its content during fermentation. In a study, Onipede et al. [176] analyzed 63 LAB strains isolated from sorghum-*ogi*, a traditional fermented cereal product, and identified *Lactiplantibacillus plantarum* W723 and *Streptococcus cremoris* W722 as the most potent phytase producers, exhibiting high phytase activity. *L. plantarum* W723 showed the highest activity at 86 U/mL, followed by *S. cremoris* W722 at 77 U/mL. Fermentation with *L. plantarum* W723 resulted in a 56.8% reduction in phytic acid content after 72 h, demonstrating its effectiveness in degrading phytate.

## 6. Applications in Fermented Beverages

Fermented beverages have become a rapidly growing segment of the food industry, especially as health-conscious consumers increasingly view them as refreshing, convenient, and nutritious, mostly due to their bioactive compound content and purported probiotic effect [20]. Fermented beverages are produced with the most diverse raw materials, including milk from various sources, such as cereals, fruits, vegetables, and leaves of tea, and fermented by specific microorganisms like LAB [177]. They are categorized, depending on their alcohol strength by volume (ABV) content, into three groups: alcoholic fermented beverages (AFB) with over 1.2% ABV, low-alcoholic fermented beverages (LAFB) with up to 1.2% ABV, and non-alcoholic fermented beverages (NAFB) with below 0.5% ABV [178]. LAB improves the quality of fermented beverages to a great level, especially those made from cereals, fruits, and vegetables. In these products, LAB fermentation can improve flavor and texture and also enhance the nutritional value due to carbohydrate hydrolysis and the release of bioactive compounds [177]. For example, LAB can ferment sugars into lactic acid, which gives a sour taste and produces an environment that is inconvenient for the growth of spoilage bacteria, thereby prolonging the shelf life of the fermented beverage [3]. In cereal-based beverages, LAB reduces antinutritional factors, such as phytic acid, by improving mineral bioavailability. Moreover, probiotics from LAB-fermented beverages also contribute to gut health; hence, they become an attractive option for health-conscious consumers [20].

### 6.1. Fermentation of Alcoholic Beverages

Alcoholic beverage fermentation, such as in beer or wine making, is a complex microbiological process involving multiple microbial transformations. While yeast is traditionally the most recognized microorganism in alcoholic fermentation, LAB also play a crucial, though often overlooked role [177]. LAB species play a crucial role in malolactic fermentation (MLF), a secondary process that occurs either during or after the primary alcoholic fermentation and significantly impacts the flavor, stability, and overall quality of the final product. MLF is a biological de-acidification process where *l*-malic acid is decarboxylated into *l*-lactic acid and CO_2_ [179]. This transformation is driven by the metabolic activity of LAB, which is present at all stages of the AFB production, such as in winemaking, and contributes to the smoothness and complexity of the final product [179]. The dynamic interaction between yeast and LAB during the production of AFB is essential for producing beverages that are not only flavorful but also stable and long-lasting. Major LAB genera involved in this process include *Leuconostoc*, *Oenococcus*, *Lactobacillus*, and *Pediococcus*, which are known for producing compounds like diacetyl, which imparts buttery flavors, as well as contributing to the release of esters and other flavor-enhancing metabolites [180]. The LAB species utilized in the fermentation of AFB contribute to the sour taste by the production of lactic acid, which lowers the pH of the environment, making it unconducive for the growth of spoilage microbes. While most LAB prefer neutral pH levels, certain strains of *Lactobacillus* and *Oenococcus* can tolerate more acidic conditions [179]. *O. oeni* is particularly well-suited for MLF in highly acidic conditions, often dominating at pH levels below 3.5, which is very unsuitable for the growth of many spoilage bacteria, thereby improving the microbial stability and shelf life of AFB [181]. Through their metabolic activities that lead to the conversion of sharp malic acid into softer lactic acid, *O. oeni* enhances the flavor profile of the beverage, creating a smoother and more balanced product [179]. On the other hand, *Pediococcus* or *Lactobacillus* strains become more prevalent at a pH above 3.5. At lower pH, glucose metabolism becomes less efficient for LAB, with species like *L. plantarum* favoring malate as an energy source, making it an effective starter for malate decarboxylation during fermentation [179,181]. This ability of *Oenococcus* and *Lactobacillus* to function at varying pH levels ensures that the fermentation process is flexible and adjustable for a range of beverage profiles while maintaining both flavor complexity and microbial stability in AFBs.

### 6.2. Non-Alcoholic and Low-Alcoholic Fermented Beverages

NAFBs and LAFBs, such as kombucha, kefir, and fermented fruit and vegetable juices, are increasingly popular in the modern food industry due to their perceived health benefits [178]. These beverages undergo a fermentation process driven by LAB alongside other microorganisms like yeast. The enzymatic activities of LAB during this process result in the formation of new bioactive compounds due to the degradation of large molecules, including carbohydrates and proteins, contributing to the development of aroma-enhancing compounds and functional molecules such as bioactive peptides, phenolic compounds, and vitamins in NAFBs and LAFBs with outstanding health benefits for humans [182,183]. For instance, LAB are highly used in berry beverage fermentations, especially for low-alcohol or non-alcoholic content. Strains such as *L. plantarum*, *L. brevis*, *L. rhamnosus*, *L. acidophilus*, and *L. casei* have already proven successful in various berry juice fermentations [17,184]. Fermentation of fruit-based beverages by LAB affects not only flavor but also the nutritional profile. During the fermentation process, LAB enables the reduction in the sugar content and, hence, decreases the alcohol content, but at the same time, increases the nutritional value by increasing the bioactive health-promoting compounds [183,184]. For example, phenolic compounds present in the berries are converted into more bioavailable states due to the action of microbial enzymes, thereby increasing the antioxidant capacity of the beverage [184]. This also contributes to the general health benefits of the beverages, such as reduced oxidative stress in the body or improved cardiovascular health [178].

Additionally, fermentation carried out by LAB enhances the flavor profiles of NAFBs and LAFBs. The microbial enzymes form esters, alcohols, and organic acids responsible for fruity, tangy, or buttery notes, depending on the type of fruit or cereal used in the process and the strain of LAB utilized [3,178,184]. Employing different strains of LAB increases the possibility of developing a variety of beverages with their own distinct flavor profile, offering more choices to meet a wide range of consumer preferences [183]. LAB also contribute to the breakdown of antinutritional factors such as phytates and tannins, which are present in fruits and vegetables, enhancing the digestibility and absorption of important minerals like calcium, iron, and magnesium in the beverages produced from them and rendering the beverages attractive to health-oriented consumers who seek nutrient-dense products [138,183]. Moreover, LAB confer probiotic properties to the NAFBs and LAFBs through the strains used in the fermentation process. LAB strains such as *L. rhamnosus* and *L. casei* that have been utilized in the production of NAFBs such as kombucha are attributed with probiotic action on gut health, immune system growth, and balanced gut microbiota [182,185].

## 7. Challenges and Future Perspectives in LAB Applications

The application of LAB in fermented foods has shown great potential in improving texture, safety, and health benefits, but this has not been without challenges. Majorly, a high number of viable cells of the probiotic LAB strains used in fermented foods need to reach the small intestine to provide their promised health effects. However, the process of food processing and storage may compromise the viability of these probiotic organisms [21]. Low pH post-fermentation, oxygen exposure during refrigeration and distribution, sensitivity to metabolites produced by other competing bacteria, and even the acidic environment of the human stomach are some factors that may compromise the survival rate of these LAB strains, thereby affecting their efficacy [21,179]. Moreover, inconsistent sensory performance of LAB-fermented products may arise due to differences in flavor and texture profiles, and this may affect consumer acceptability [29]. Also, while many LAB species have been generally recognized as safe for use in food production, the presence of antimicrobial resistance genes and virulence factors in species from the *Enterococcus* genus have raised food safety concerns, as they can mediate gene transfer with different genetic elements. Due to this, the *Enterococcus* genus has not yet been classified as safe for human consumption nor granted GRAS status [94].

In addressing these challenges, improved methodologies of microencapsulation have been developed to protect bacterial cells against damage from the external environment. LAB strains can be encapsulated to protect them from pH, oxygen exposure, or stomach acid and enhance survival and persistence in the human gastrointestinal tract [186]. However, these solutions are often strain-specific, indicating that each probiotic LAB strain may require a tailored approach to ensure optimal survival [187]. The regulatory features for the use of LAB in food are still a huge challenge as there are very rigid rules to label LAB as a probiotic or natural preservative in food products, which has tremendously restricted the ability of manufacturers to make specific health claims for their products [188]. Also, while many consumers are increasingly aware of the benefits of probiotics, further education on LAB and their benefits in food preservation and health is still required. Misconceptions or lack of knowledge about the products inform purchasing decisions; hence, there needs to be a balance between marketing strategies by food producers and more transparent communications regarding the science behind the benefits of LAB.

Currently, different areas show great potential in advancing the role of LAB in food technology, health, and sustainability. This includes genetic and metabolic engineering, which focuses on genetically modifying LAB strains to improve or introduce new functionalities, such as producing higher quantities of beneficial metabolites like organic acids, bacteriocins, or exopolysaccharides. In this regard, genetic engineering has aimed at accelerating cheese ripening, for instance, by developing strains with environmentally controlled lytic behavior [189]. When induced, these kinds of strains release more quantities of their cytoplasmic enzymes into the cheese matrix with consequent acceleration in ripening. This enzymatic release increases peptide hydrolysis, which indirectly enhances aroma by increasing the pool of free amino acids available for metabolism by other cells present in the cheese [189]. This area requires further exploration as it has the potential to contribute immensely towards the utilization of LAB in engineering the organoleptic profile and acceptance of many foods.

### 7.1. Strain Selection and Stability

The selection and stability of strains are two important success factors to consider when applying LAB in food and beverages. Traditionally, LAB have been used in a wide range of fermented foods and beverages to enhance flavor, texture, and shelf life and for their probiotic health benefits. The challenge is in the selection of appropriate LAB strains with different product applications and also in their stability during production and storage. The immense variability in the various products and environments in which they are expected to function poses a challenge when selecting LAB strains for use [21]. The composition, pH, water activity, and conditions of processing for fermented foods and beverages vary greatly and can affect the growth, metabolic activity, and viability of the strains of LAB [22]. It is, therefore, important to select strains that will perform well under product conditions. For example, LAB strains that are effective in dairy fermentation may not act quite as effectively in plant-based or fruit-based fermentations due to the extreme difference in nutrient availability and environmental conditions [187]. The nutritional requirements of LAB strains often reflect the ecology in which they were adapted; for instance, strains like *L. plantarum* from plant sources usually exhibit fewer auxotrophies, with extended biosynthetic capabilities compared to *L. johnsonii* from the nutrient-rich human gastrointestinal tract [187,190]. Understanding these nutritional requirements and designing specific fermentation media for specific strains is crucial for their optimal growth, survival, and performance during the production process.

Apart from the product-related requirements, LAB strains must also exhibit certain functional properties that must be considered for use in food production. These traits include the ability to generate desirable flavor compounds, enhance texture, and inhibit spoilage microorganisms [3,29]. In addition, strains with probiotic potential should survive the passage through the human gastrointestinal tract, especially the acidic condition of the stomach, to be able to exert beneficial properties to the host. This complication further narrows the selection of strains since only a few strains possess technological as well as probiotic properties that are required by a specific product [21]. Furthermore, LAB viability is interfered with at each stage in the production process with temperature variability, oxygen uptake, and the presence of other microorganisms [21,191]. For instance, the optimal growth temperature for most LAB strains ranges between 30 and 45 °C, with some thermophilic strains, such as *Lactobacillus acidophilus* and *Streptococcus thermophilus*, able to thrive at temperatures up to 45 °C [192]. However, temperatures outside this range, particularly lower temperatures, subject LAB to thermal stress, reducing their growth and viability.

Notwithstanding these challenges, the past two decades have seen phenomenal advancement in strain selection and stability. Microencapsulation techniques have shown promise in protecting the LAB cells from environmental stresses in the processes of production and storage, although large variability concerning their activities among strains and applications exists, emphasizing the need for further research to be conducted to optimize this technique for different food matrices [186]. Novel LAB strains are constantly being studied to unravel those that exhibit better resistance to various types of stressors such as heat, oxygen, and low pH. Further genetic engineering and bioengineering might also enhance robustness and performance in various food matrices by re-designing the existing LAB strains.

### 7.2. Regulatory Aspects and Consumer Acceptance

Despite the numerous benefits of LAB, regulatory challenges and consumer perceptions continue to influence the integration of LAB into the food market, especially when it comes to labeling them as probiotics or natural preservatives. For a microorganism to be declared and sold as a probiotic, it has to meet the required criteria involving health claims and safety parameters drawn up by the various regulatory authorities in different parts of the world: the FAO/WHO, the Food and Drug Administration (FDA) in the United States, the European Food Safety Authority (EFSA) in Europe, among others [23,193]. These authorities want solid scientific proof for the claims of health benefits for a particular LAB strain, which will probably include several clinical trials, studies of the strain concerned, and safety assessments, all of which are time-consuming and costly. Additionally, these LAB strains should meet certain specifications related to viability and safety as probiotics, such as the ability to withstand bile acid, adhere to human epithelial cells, resist gastric juice, decrease pathogens in the gut, exhibit antimicrobial activity, inhibit bile salt hydrolase activity, and to assess safety [194]. Apart from using them as probiotics, there is an increasing awareness of their application as natural preservatives in maintaining product quality. The growth of spoilage microorganisms in food is inhibited by organic acids, bacteriocins, and other antimicrobial compounds produced by LAB, extending its shelf life [3]. Despite these benefits, regulatory guidelines for labeling LAB as natural preservatives are still underdeveloped globally. While bacteriocins produced by LAB, such as *L. lactis* nisin, have gained acceptance along with approvals to be used as preservatives in certain regions, gaining approvals for new compounds derived from the LAB is an elaborate process [24]. Key concerns that must be addressed before incorporating LAB as natural antimicrobials in food products include their cost, sensory impact, regulatory status, potential toxicological and allergenic effects, interaction with food ingredients, proven effectiveness, and lack of harmful effects on the natural microflora [94].

Growing consumer awareness and demand for clean-label ingredients have led to increasing skepticism toward artificial additives and preservatives, driving interest in natural alternatives like LAB [23]. The use of LAB aligns well with the clean-label movement, which promotes products with simple, easily recognizable ingredients, since LAB are naturally occurring and, therefore, generally perceived as a healthier alternative to synthetic additives [195]. However, the choice of carriers and ingredients in the development of functional foods plays a crucial role in shaping consumer perceptions and acceptance, as these components influence not only the product’s taste, texture, and appearance but also its health benefits [196]. Consumers increasingly seek out products that offer natural and recognizable ingredients, which they associate with safety, quality, and overall wellness. Therefore, the level of consumer acceptance can be influenced by the transparency of product labeling, the functionality of the ingredients, and the alignment with consumer values such as sustainability and healthfulness [196,197]. Consumers also tend to be skeptical about the methods employed in the incorporation of these functional carriers into food products as they are more likely to prefer functional foods where the enrichment of ingredients occurs naturally or through traditional processes, as this aligns with their desire for authenticity and minimal processing [196,198]. Therefore, there exists a need for guidelines on more clearly defined labeling and consumer education campaigns to narrow the gap between scientific knowledge and public understanding. Consumer-friendly labeling may be critical in this process. If food companies could explain the role of LAB in the preservation of food or gut health, they would make the process less mystifying for consumers. Indeed, clear information about the safety and efficacy of LAB is going to be transparently communicated in an effort to build consumer acceptance, considering that more consumers are looking toward natural and probiotic-containing products for health benefits.

### 7.3. Innovation in LAB Fermentation

In light of the increasing adoption of plant-based diets and demand for sustainable and health-conscious products, fermentation with LAB has steadily been on the rise. Traditionally used in manufacturing dairy, fermented vegetables, and alcoholic beverages, LAB are being put to other uses in new areas of food processing to meet the developing range and functionality of fermented foods. One major area is the use of LAB for fermentation of plant-based food products, which has significantly grown with increasing vegetarian and vegan diets to create healthy, flavorful, and shelf-stable products without the use of animal-based ingredients [199]. Today, LAB are employed to ferment a wide array of materials in plants such as soy, almond, oats, coconut, and other legumes and grains. LAB fermentation not only preserves these food products but also enhances their nutritional profiles due to the generation of bioactive compounds, vitamins, and probiotics, besides degrading anti-nutrients like phytates and lectins [17]. While these plant-based products are excellent sources of a variety of vitamins, they generally contain low levels of some highly important vitamins for human health, such as vitamins B and K [199]. In addition to the advantageous acidic environment created by LAB fermentation, which preserves and improves the stability of existing vitamins in these products, it also promotes additional synthesis of vitamins [17]. LAB fermentation will enable natural and sustainable production of vitamin K directly in the food matrix. The study by Leksono et al. [200] demonstrated the ability of LAB to enhance the antioxidant properties of black soy milk during its fermentation. In the study, all three LAB strains (*Lactiplantibacillus plantarum* WGK 4, *Streptococcus thermophilus* Dad 11, and *Lactiplantibacillus plantarum* Dad 13) that were used improved antioxidant activity from 24.90% to 31.22–38.20%, followed by an increase in isoflavone aglycone, demonstrating the potential of LAB to be used as a starter culture in the fermentation of plant-based products to improve their nutritional value.

Nowadays, scientists try to genetically modify or select LAB strains with desirable improved traits, including enhanced probiotic potential, higher tolerance against stressors during production and storage, or the production of certain compounds that improve health, such as vitamins or enzymes [201]. Other modifications could include bioengineering of LAB strains to enhance the production of bioactive compounds, including bacteriocins, which are naturally occurring antimicrobial compounds used to extend the shelf life of foods, or improving the ability of LAB to survive under extreme environmental conditions, such as high temperatures during food processing or low pH in food matrices [202]. These technological developments have the potential to ensure greater survival rates of LAB in the final product, which in turn allows for more consistent delivery of both probiotics and health benefits to the consumer. Despite these efforts, only a few LAB bacteriocins have reached commercial application: nisin, a lantibiotic peptide produced by *L. lactis*; pediocin PA-1 from *Pediococcus acidilactici*; and carnocyclin A from *Carnobacterium* maltaromaticum UAL307 [203]. These bacteriocins have been the focus of bioengineering efforts aimed at enhancing their effectiveness in food systems. Recently, bioengineered derivatives of nisin have shown improved efficacy, particularly in complex food matrices. Newly developed nisin variants in the study by Rouse et al. [204] demonstrated an enhanced ability to diffuse through polymers, outperforming nisin A in inhibiting *Listeria monocytogenes* growth in commercial chocolate milk containing carrageenan as a stabilizer, reflecting the potential of bioengineering to improve the functionality of LAB bacteriocins in food preservation. LAB fermentation fits well within the increasingly popular notion of food production sustainability, and hence, its combination with other emerging technologies, such as precision fermentation and synthetic biology, may lead to even more opportunities. These emerging developments will also very likely include added-value LAB strains tailored for specific nutritional or functional needs, which again will extend the health and sustainability dimensions of fermented foods.

Although genetic modification of LAB does offer promising avenues to improve food properties, application in food products is significantly limited. The regulatory challenges are huge since most regions have legislation imposing strict approval and labeling for genetically modified organisms (GMOs), causing delays in the development process and limiting market access for these strains [189]. Moreover, low acceptance of consumers to genetically engineered LAB is common; the generally low perceived characteristics of safety and naturalness lower consumer acceptance and demand for such foods [23]. Additionally, maintaining the stability and functionality of the introduced genetic traits in complex food environments is difficult; genetically engineered traits may not always perform consistently during large-scale production and extended storage [189]. In all, these factors make the application of genetically modified LAB difficult to spread widely in food production and urge researchers to find alternative approaches that can keep their work within the requirements set by regulatory authorities and consumers.

### 7.4. Sustainability

The global food system is under increasing pressure to meet the needs of a growing population while minimizing its environmental footprint. Food waste is a significant contributor to this problem, with an estimated one-third of all food produced globally being wasted, leading to economic losses, contribution to greenhouse gas emissions, resource depletion, and food insecurity [205]. Over the past years, LAB have become a promising route to address these challenges by providing new methods of reducing food loss as well as promoting sustainable food production [25]. One of the major reasons for the widespread adoption of LAB fermentation is its ability to extend the shelf life of perishable foods by producing lactic acid, which lowers the pH and inhibits the growth of spoilage-causing microorganisms [3]. This simple yet effective process helps preserve easily spoiled foodstuffs, making it a key method for reducing food waste. This process serves as a natural preservative technique for fermented food products such as yogurt, sauerkraut, kimchi, and pickled vegetables, enabling them to be stored for a long duration [2,55]. LAB fermentation allows producers to transform surplus or cosmetically defective fruits and vegetables into valuable products like pickles or sauces, reducing food waste and increasing revenue [33]. This process promotes both economic sustainability and environmental protection by preventing edible food from being discarded. In regions where storage by refrigeration is too expensive or simply unavailable, food preservation by fermentation provides an effective alternative to reduce losses on foods that spoil shortly after harvest.

Sustainability in food production also involves the reduction in synthetic chemicals, which can have severe consequences in terms of health and environmental impact [206]. LAB fermentation represents a natural alternative to chemical preservatives and additives that are used so often in a wide variety of food products [23]. Since LAB can form natural antimicrobial compounds, such as bacteriocins, that can inhibit foodborne pathogens, it therefore reduces the need for chemical preservatives. Nisin, a bacteriocin produced by *L. lactis*, has already found practical application as a natural food additive that acts as a preservative for food products [24]. In extending the use of LAB-derived compounds, food industries will be less dependent on chemical preservatives since such foods are safer to eat and more stable, thereby fostering organic farming and organic processing and, hence, sustainability-related objectives. Besides their present contribution to food preservation and reduction in food losses, LAB also play an active role in sustainable agriculture. LAB are involved in the preparation of fermented compost and silage that improve soil fertility and crop yield with little or no use of synthetic fertilizers and pesticides [206]. In silage fermentation, LAB play a key role by rapidly producing low pH levels, which help preserve the feed. By dominating the fermentation process, LAB allow animal feed to be stored for extended periods without spoilage, ensuring a consistent and nutritious supply of food for livestock, even during low-production agricultural seasons [207]. This decreases the reliance on imported feed that normally comes with great environmental costs. Furthermore, LAB can be applied as probiotics in animal husbandry to enhance gut health and immune function in livestock, which will reduce the use of antibiotics and, hence, help combat antimicrobial resistance [208]. This is particularly important in sustainable farming systems, where reducing antibiotic use is a key priority. Using this approach, LAB improves animal health and productivity, therefore opening ways toward more eco-friendly means of farming without compromising food security.

## 8. Conclusions

LAB have been shown to be multi-functional microorganisms whose roles have ranged from being considered essential for fermentation and preservation to the enhancement of nutritional value, food safety, and even probiotic functions. It is, therefore, well established that these bacteria are critically important in modern food systems. Hence, diversifying the applications of LAB into novel food and beverage products, such as plant-based alternatives and bioengineered strains with enhanced functionalities, holds great potential for innovative developments in the field of sustainable food production. Furthermore, continued exploration of LAB strains for specific health benefits, environmental sustainability, and food waste reduction will shape different industries, especially the food industry. With LAB at the forefront, in response to the consumers’ demand for clean-label products coupled with improvement in bioengineering techniques, it is well set to remain one of the most important agents of change in modern food technology for the promotion of global health. With constant research and innovation, their role in the development of healthy and sustainable food systems is bound to become even more critical in the future. In any case, the full realization of the potential of LAB in sustainable food systems will need consumer education and regulatory support. Growing consumer demand for more natural and sustainable foods will increasingly look toward LAB as a clean-label ingredient that is friendly to the environment. In this regard, policymakers can make the use of LAB in food production much easier by giving clear guidelines and thus encouraging research into their benefits.

## Figures and Tables

**Table 1:** Common types of LAB and their scope of application.

Type of LAB	Scope of Application	References
*Lactobacillus*	Dairy fermentation (yogurt, cheese, kefir), probiotic supplementation, and fermentation of vegetables.	[3,26]
*Lactococcus*	Development of flavor and texture in fermented foods, fermentation of dairy-based products, and biopreservative constituents.	[1,26]
*Leuconostoc*	Improvement of flavor and aroma in fermented vegetables	[1,26]
*Pediococcus*	Fermentation of beer and wine, improvement of taste in fermented beverages, and inhibition of pathogenic microorganisms in fermented food products.	[1,26]
*Streptococcus*	Production of yogurt and cheese	[1,26]
*Oenococcus*	Malolactic fermentation in wine, improving flavor and stability	[1]
*Weissella*	Fermentation of vegetables, fish, and other traditional foods	[1]
*Enterococcus*	Improvement in the aroma, texture, and flavor of fermented dairy products	[1]

**Table 2:** Metabolites synthesized by LAB and their primary functions.

Metabolite	Type	Function(s)	Synthesizing Organism	References
Organic acids	Lactic, acetic, gamma-aminobutyric, and linoleic acids	Lowers pH, inhibits spoilage/pathogenic microbes	*L. plantarum*, *L. casei*, *L. acidophilus*	[3,44]
Bacteriocins	Class I, class II, class III, and class IV	Inhibits the growth of pathogenic bacteria	*L. lactis*, *L. plantarum*	[42,44]
Vitamins	Riboflavin (B2), folic acid (B9), cobalamin (B12), vitamin K.	Enrichment of food’s nutritional value	*L. lactis*, *L. plantarum*	[44]
Exopolysaccharides	Homopolysaccharides or heteropolysaccharides	Enhances texture and increases viscosity	*L. plantarum*, *L. bulgaricus*, *L. reuteri*	[13,45,46]
Enzymes	Proteases, β-galactosidase	Aids in the breakdown of carbohydrate, protein and lactose	*L. acidophilus*, *L. helveticus*	[46]

## Data Availability

No new data were created or analyzed in this study. Data sharing is not applicable to this article.
